# Systematic reviews followed by clinical trials, followed by systematic reviews: this is how the uncertainties in medicine are reduced

**DOI:** 10.1590/S1516-31802007000200001

**Published:** 2007-03-04

Around 20 years ago, we started some work on evidence-based medicine that our medical colleagues called "working like ants building anthills". We began with talks and educational articles on the importance of clinical epidemiology for improving clinical research and, at the same time, we emphasized the importance of systematic reviews for human health.^1,2^ In parallel, we conducted prospective cohort studies on hypertensive pregnant women that showed the serious situation regarding chronic hypertension during pregnancy within our environment. Whereas gestational hypertension is taken in the first world to have few clinical consequences, here it has been associated with severe maternal-fetal complications in almost 60% of the cases.^3^

On the basis of this study, we carried out a large clinical trial on the use of low doses of aspirin (80 mg/day) for preventing preeclampsia and its consequences.^4^ This trial included 1000 patients, of whom 500 were hypertensive and showed that aspirin had no major effect regarding the prevention of preeclampsia, contrary to what had been suggested by articles published in the leading journals Lancet^5^ and New England Journal of Medicine.^6^ In parallel, we developed the Eclampsia Trial with colleagues in Oxford and Latin American and African countries. This showed that the effectiveness of the traditional remedy magnesium sulfate was greater than the effectiveness of all the drugs launched and adopted over the 90 years that followed the initial use of magnesium sulfate. The Eclampsia Trial changed the way in which convulsions due to eclampsia were treated in countries where more than three billion people live. It was considered to be the best clinical trial of the twentieth century in editorials published by leading medical journals.^7,8^ Today, it is the standard treatment for convulsions due to eclampsia.^8,9^ In subsequent work by the same group (the Magpie Trial), it was found that administration of magnesium to pregnant women with hypertension and proteinuria reduced the chance that the patients would develop eclampsia by 67%.^10^ It should be added, incidentally, that magnesium sulfate is extremely inexpensive and is a substance that forms a large proportion of the earth’s crust!

A systematic review conducted by us and colleagues in South Africa (Justus Hofmeyr) and the United Kingdom (Lelia Duley) in 1998^11^ showed the possible benefits of calcium supplementation in preventing preeclampsia and maternal-fetal morbidity among pregnant women with low calcium intake (calcium-poor) who were at high risk of preeclampsia.

On the basis of this review, the World Health Organization (WHO) started to recommend calcium supplementation for the diet of pregnant women. There is no evidence of risks. More recently, WHO itself funded a mega-trial to compare calcium supplementation during pregnancy with placebo, among low-risk patients, which also showed that calcium supplementation decreased the incidence of preeclampsia and maternal-fetal morbidity.^12^ With the data from this mega-trial, we improved the systematic review that was initially published in the Cochrane Library and submitted it for publication in the British Medical Journal (Hofmeyr J, Atallah AN and Duley L), where it has been accepted and is awaiting publication. Through this, practically all pregnant women around the world will be advised to adopt a calcium-rich diet (or to take calcium carbonate, between 500 mg/day and 2,000 mg/day), in order to prevent hypertensive complications during pregnancy. Also recently, the PARIS group, constituted by colleagues who took part in the abovementioned clinical trials, met in Oxford to discuss the systematic review on the use of aspirin in low doses for preventing preeclampsia. The meta-analyses included are the Collaborative Study for Prevention of Preeclampsia with Aspirin (*Estudo Colaborativo para Prevenção da Pré-eclâmpsia com Aspirina*, ECPPA)^4^ and data on more than 29,000 cases that had been included in clinical trials on this subject. This systematic review, which brings together data on the individual cases of around 30,000 patients and has been accepted for publication by the Lancet, is a model of what we can call the quintessence of evidence: it defines the effect. It should be compared with the systematic review with meta-analysis that ECPPA published in 1996^4^ and with the other review that was published in the Cochrane Library.^11^ Through this, the uncertainties on the subject are being progressively reduced, in an appropriate manner.

Thus, over the last 20 years, we have learned much about how to obtain evidence from research with adequate methodology and through systematic reviews of the literature. It can be said in passing that all the studies cited here were preceded by systematic reviews; the study on aspirin was preceded and complemented by a systematic review. All this learning was shared with undergraduate and postgraduate students and with practicing professionals and medical entities through the Scientific Board of the São Paulo Medical Association (*Associação Paulista de Medicina*, APM), and also with healthcare professionals in all fields.

In October of this year, many stories like these and the consequent evidence obtained will be presented at the Fifteenth Cochrane Colloquium, "Evidence-Based Healthcare for all", which is to be held in São Paulo from October 23 to 28, 2007. All perspectives, whether from patients, doctors, healthcare professionals, judges and attorneys, managers responsible for healthcare companies, health departments of states and municipalities, health ministries of dozens of countries, or international entities like WHO and the Pan-American Health Organization (PAHO), will be integrated for the benefit of the best evidence in healthcare for all. Mark it in your diaries: http://www.colloquiumbrasil.info/ — www.centrocochranedobrasil.org.
